# Supplementary material for: The effect of antidepressants on the severity of COVID-19 in hospitalized patients: A systematic review and meta-analysis
Source: PLoS One. 2022 Oct 6;17(10):e0267423. doi: 10.1371/journal.pone.0267423 (PMC9536564; doi:10.1371/journal.pone.0267423)
Supplement: S3 File — (DOCX) [file pone.0267423.s003.docx]

| #1 | "antidepressive agents"[MeSH Terms] OR "antidepressive agents, second generation"[MeSH Terms] OR "antidepressive agents, tricyclic"[MeSH Terms] OR "monoamine oxidase inhibitors"[MeSH Terms] OR "serotonin and noradrenaline reuptake inhibitors"[MeSH Terms] OR "serotonin uptake inhibitors"[MeSH Terms] OR "SSRI"[Title/Abstract] OR "SNRI"[Title/Abstract] OR "TCA"[Title/Abstract] OR "MAOI"[Title/Abstract] OR "Antidepressive"[Title/Abstract] OR "Anti-depressive"[Title/Abstract] OR "Anti-depression"[Title/Abstract] OR "Antidepression"[Title/Abstract] |
| --- | --- |
| #2 | "COVID-19"[MeSH Terms] OR "COVID-19"[Title/Abstract] OR "SARS-CoV-2"[MeSH Terms] OR "SARS-CoV-2"[Title/Abstract] OR "2019 novel coronavirus"[Title/Abstract] OR "sars coronavirus 2"[Title/Abstract] |
| #3 | ("antidepressive agents"[MeSH Terms] OR "antidepressive agents, second generation"[MeSH Terms] OR "antidepressive agents, tricyclic"[MeSH Terms] OR "monoamine oxidase inhibitors"[MeSH Terms] OR "serotonin and noradrenaline reuptake inhibitors"[MeSH Terms] OR "serotonin uptake inhibitors"[MeSH Terms] OR "SSRI"[Title/Abstract] OR "SNRI"[Title/Abstract] OR "TCA"[Title/Abstract] OR "MAOI"[Title/Abstract] OR "Antidepressive"[Title/Abstract] OR "Anti-depressive"[Title/Abstract] OR "Anti-depression"[Title/Abstract] OR "Antidepression"[Title/Abstract]) AND ("COVID-19"[MeSH Terms] OR "COVID-19"[Title/Abstract] OR "SARS-CoV-2"[MeSH Terms] OR "SARS-CoV-2"[Title/Abstract] OR "2019 novel coronavirus"[Title/Abstract] OR "sars coronavirus 2"[Title/Abstract]) |

Table S1. Search strategy of PubMed/Medline

| #1 | 'antidepressant agent'/exp OR 'tricyclic antidepressant agent'/exp OR 'monoamine oxidase inhibitor'/exp OR 'serotonin uptake inhibitor'/exp OR 'serotonin noradrenalin reuptake inhibitor'/exp OR ssri:ti,ab,kw OR snri:ti,ab,kw OR tca:ti,ab,kw OR maoi:ti,ab,kw OR antidepressive:ti,ab,kw OR 'anti depressive':ti,ab,kw OR antidepression:ti,ab,kw OR 'anti depression':ti,ab,kw |
| --- | --- |
| #2 | 'coronavirus disease 2019'/exp OR 'severe acute respiratory syndrome coronavirus 2'/exp OR 'covid 19':ti,ab,kw OR 'sars-cov 2':ti,ab,kw OR 'sars cov 2':ti,ab,kw OR 'sars cov2':ti,ab,kw |
| #3 | ('antidepressant agent'/exp OR 'tricyclic antidepressant agent'/exp OR 'monoamine oxidase inhibitor'/exp OR 'serotonin uptake inhibitor'/exp OR 'serotonin noradrenalin reuptake inhibitor'/exp OR ssri:ti,ab,kw OR snri:ti,ab,kw OR tca:ti,ab,kw OR maoi:ti,ab,kw OR antidepressive:ti,ab,kw OR 'anti depressive':ti,ab,kw OR antidepression:ti,ab,kw OR 'anti depression':ti,ab,kw) AND ('coronavirus disease 2019'/exp OR 'severe acute respiratory syndrome coronavirus 2'/exp OR 'covid 19':ti,ab,kw OR 'sars-cov 2':ti,ab,kw OR 'sars cov 2':ti,ab,kw OR 'sars cov2':ti,ab,kw) |

Table S2. Search strategy of Embase

| #1 | TITLE-ABS-KEY ( antidepressive AND agent ) OR TITLE-ABS-KEY ( antidepressive AND agents, AND second AND generation ) OR TITLE-ABS-KEY ( antidepressive AND agents, AND tricyclic ) OR TITLE-ABS-KEY ( monoamine AND oxidase AND inhibitors ) OR TITLE-ABS-KEY ( serotonin AND noradrenaline AND reuptake AND inhibitor ) OR TITLE-ABS-KEY ( serotonin AND uptake AND inhibitor ) OR TITLE-ABS-KEY ( ssri ) OR TITLE-ABS-KEY ( snri ) OR TITLE-ABS-KEY ( tca ) OR TITLE-ABS-KEY ( maoi ) OR TITLE-ABS-KEY ( antidepressive ) OR TITLE-ABS-KEY ( anti-depressive ) OR TITLE-ABS-KEY ( antidepression ) OR TITLE-ABS-KEY ( anti-depression ) OR TITLE-ABS-KEY ( antidepressant AND agent ) OR TITLE-ABS-KEY ( tricyclic AND antidepressant AND agent ) |
| --- | --- |
| #2 | TITLE-ABS-KEY ( covid-19 ) OR TITLE-ABS-KEY ( sars-cov-2 ) OR TITLE-ABS-KEY ( 2019 novel AND coronavirus ) OR TITLE-ABS-KEY ( sars AND coronavirus 2 ) OR TITLE-ABS-KEY ( coronavirus AND disease 2019 ) OR TITLE-ABS-KEY ( severe AND acute AND respiratory AND syndrome AND coronavirus 2 ) |
| #3 | ( TITLE-ABS-KEY ( antidepressive AND agent ) OR TITLE-ABS-KEY ( antidepressive AND agents, AND second AND generation ) OR TITLE-ABS-KEY ( antidepressive AND agents, AND tricyclic ) OR TITLE-ABS-KEY ( monoamine AND oxidase AND inhibitors ) OR TITLE-ABS-KEY ( serotonin AND noradrenaline AND reuptake AND inhibitor ) OR TITLE-ABS-KEY ( serotonin AND uptake AND inhibitor ) OR TITLE-ABS-KEY ( ssri ) OR TITLE-ABS-KEY ( snri ) OR TITLE-ABS-KEY ( tca ) OR TITLE-ABS-KEY ( maoi ) OR TITLE-ABS-KEY ( antidepressive ) OR TITLE-ABS-KEY ( anti-depressive ) OR TITLE-ABS-KEY ( antidepression ) OR TITLE-ABS-KEY ( anti-depression ) OR TITLE-ABS-KEY ( antidepressant AND agent ) OR TITLE-ABS-KEY ( tricyclic AND antidepressant AND agent ) AND TITLE-ABS-KEY ( covid-19 ) OR TITLE-ABS-KEY ( sars-cov-2 ) OR TITLE-ABS-KEY ( 2019 novel AND coronavirus ) OR TITLE-ABS-KEY ( sars AND coronavirus 2 ) OR TITLE-ABS-KEY ( coronavirus AND disease 2019 ) OR TITLE-ABS-KEY ( severe AND acute AND respiratory AND syndrome AND coronavirus 2 ) ) |

Table S3. Search strategy of Scopus
