# Supplementary material for: The effect of antidepressants on the severity of COVID-19 in hospitalized patients: A systematic review and meta-analysis
Source: PLoS One. 2022 Oct 6;17(10):e0267423. doi: 10.1371/journal.pone.0267423 (PMC9536564; doi:10.1371/journal.pone.0267423)
Supplement: Attachment — Submitted filename: plos-one.docx [file pone.0267423.s004.docx]

The effect of antidepressants on severity of COVID-19 in hospitalized patients: A systematic review and meta-analysis

Thank you for inviting me to forward my comments regarding the manuscrit. The title is attractive, however the review and analysis is shallow. I have the following major concerns

1. It is not advisable to include abbrivations in the abstract
2. You have used 3 different quality assement methods. How can you assess the over all quality of the manuscript using different tools?
3. In the methods part stated that heterogeneity was assessed by Cochran’s Q and the I2 statistic. Publication bias was evaluated statistically by using Egger’s and Begg’s tests. However, there is no values in the pooled analysis stating the findings (Q and I^2^) and no egger’s and Begg’s test.
4. If so how do you select the fixed model?
5. Systematic review and meta analysis is recommended if the study designes are similar. Other wise, mixing different protocols gives un nessary interpretation.
