# Supplementary material for: The effect of antidepressants on the severity of COVID-19 in hospitalized patients: A systematic review and meta-analysis
Source: PLoS One. 2022 Oct 6;17(10):e0267423. doi: 10.1371/journal.pone.0267423 (PMC9536564; doi:10.1371/journal.pone.0267423)
Supplement: Attachment — Submitted filename: Response to Reviewers.docx [file pone.0267423.s005.docx]

We thank the editor and the reviewers for their comments on our manuscript. Below is our response to each point raised by the academic editor and reviewers. We hope that we satisfyingly addressed them and that the manuscript will be now suited for publication.

Sincerely,

On behalf of all authors,

**Moein Zangiabadian**

**Dear editor**

Thank you for considering our manuscript. We thank the editor and reviewers for their thoughtful critique and comments. We have carefully edited the manuscript as requested by you and have provided a point-by-point response below. Please find the revised version included. The revisions are highlighted in yellow in the resubmitted manuscript. We hope this meets the established reputation for the quality of your esteemed journal.

1) Thanks for pointing this out. The manuscript is updated with PLOS ONE's style requirements.

2) Thanks for your fair and constructive comment. The duplicate text in discussion is rephrased word by word.

3) Thanks for pointing this out. The supplementary file (excel of data extraction) for data availability statement is uploaded and the sentence of this section is changed. (Page 15: line 576)

4) Thanks for pointing this out. The ORCID iD is added in Editorial Manager.

5) Thanks for pointing this out. The tables S1&S2&S3 are uploaded as supplementary file (search strategy).

**Dear reviewer 1**

We appreciate the time and attention you spent in reviewing our manuscript.

1) Our manuscript is a systematic review of the preventive effect of antidepressants on COV-19 infections and we have discussed about different types of antidepressants in our manuscript and the meta-analysis of the effect of fluvoxamine on COVID-19 is only a part of our results not all of them. Your two mentioned studies are only about the fluvoxamine and have not discuss about other antidepressants. The first study (PMID: 35385087) has included three RCTs for meta-analysis and one of them (*Lenze E. Fluvoxamine for early treatment of COVID-19: a fully-remote, randomized placebo controlled trial*) was not in our analysis so we added it. On the other hand, one of our included studies (Calusic M. Safety and efficacy of fluvoxamine in COVID-19 ICU patients: An open label, prospective cohort trial with matched controls) was not in their analysis so the new analysis with **five** studies that we have performed, is updated in this subject. The second study (PMID: 35383578) has included three studies with different methods (two RCTs and one prospective cohort) and has analyzed them but this is not correct to analyze interventional and observational studies together. The three included articles of this study are common with our included studies.

2) There were diversity in study designs and type of antidepressants and we only could analyze the clinical trials about the effect of fluvoxamine. So we did not restrict our results to this analysis and systematically reviewed the effect of other antidepressants in studies with different methods. Also the analysis is updated and **five** studies are included.

**Dear reviewer 2**

Thank you for your willingness to consider our initial manuscript “The effect of antidepressants on severity of COVID-19 in hospitalized patients: A systematic review and meta-analysis”. We have carefully considered all comments and revised and improved some parts of the original manuscript as requested. We hope this meets the established reputation for the quality of your esteemed journal. The revisions are highlighted in yellow in the resubmitted manuscript.

1) Thanks for your fair and constructive comment. The search is updated till June 14, 2022, and two further studies (RCTs) about the effect of fluvoxamine on COVID-19 are added. But your suggested article (doi: 10.1093/ofid/ofac156. PMID: 35531374) about fluoxetine didn’t meet the inclusion criteria. Because the aim of our study is to review the articles which evaluate the effect of antidepressants on reducing the severity of Covid-19 and MacFadden et al did not compare this aforementioned goal and focused on infection risk and detection of SARS-CoV-2, so this article was excluded. (Page 3: line 113)

2) Thanks for pointing this out. As it is shown in table 1, all included studies for meta-analysis have used PCR test for detection of COVID-19. So there is no concern about your mentioned bias.

3) Thanks for your recommendation. The analysis adjustment for your mentioned factors is extracted from all included studies and added in table 5 and unfortunately most studies have not adjust their results by these factors so it can cause bias and we added this point to our study limitations in discussion section (Page 13: lines 524-531). On the other hand, among included clinical trials that we have analyzed, only one of them is not randomized control trial (Calusic et al.) but this study has mentioned that there was matching between two groups in the basis of age, sex, co-morbidities, vaccination status and disease severity (see table 5) so there is no concern about this study although in the quality assessment we mentioned this study with high risk of bias.

4) Thanks for your suggestion. Study characteristics like study period and country are in table 1.

5) Thanks for your recommendation. Unfortunately it’s not possible to Meta-analyze any antidepressant or molecule because there are not enough studies (at least three studies) in the same design to analyze them together. For example, there are two cohorts and one case control study about fluoxetine or one cohort and two case control studies about escitalopram. On the other hand, the data is common between SSRIs and SNRIs in three studies and there are not separate data for each of them for analyzing.

6) Thanks for your suggestion. As it mentioned in above comment, we only analyzed the clinical trials and the I2 for our analysis was 0.0 so the heterogeneity is very low and there was not any conflicting results in our analysis so sensitivity analysis is not necessary.

7) Thanks for pointing this out. Table 4 is changed and quality assessment section is corrected. (Page 5: lines 176-179)

8) Thanks for your suggestion. The primary outcome is mentioned in result section as a significant difference between two groups but unfortunately the raw data about this outcome is not available for analysis and we used secondary outcome raw data for our analysis. (Page 7: lines 251-258)

9) Thanks for your recommendation. The primary or main outcome in this study was not clarified and all mentioned outcomes were secondary and statistically insignificant. On the other hand, all data in Calusic et al. study were continuous except the data about mortality that was dichotomous and we used of this outcome for analysis.

10) Thanks for your fair and constructive comment. All of your comments are correct but as we have added in table 6 this study is adjusted for comorbidities. However,table 3 and quality assessment section is changed according to the McKeigue et al. high risk of bias and the discussion is balanced. (Page 5: lines 176-179, page 8: lines 287-290)

11) Thanks for pointing this out. Tables 2 and 6 are corrected.

12) Thanks for your fair and constructive comment. Discussion is corrected in regard of your suggestions:

- “Revision of following sentence « This could explain the findings of Hoertel et al. study (8), although considering that there are other drugs such as chlorpromazine with ASM inhibition activity that didn’t show the same protective effects on mortality in COVID-19 patients, this mechanism is unlikely a major factor for said effect”, and 3 factors including (i) the magnitude of the in vitro effect of the ASM (ii) the dose prescribed, and (iii) the concentration in the lungs” are added. (Page 11: lines 414-436)
- “Chlorpromazine inhibition of ASM in in-vitro condition” is added. (Page 11: lines 422-426)
- “Observational studies which show that inhibition of acid sphingomyelinase/ceramide as a potentially important role” is added. (Page 10: lines 396-401)
- “Both potential antiviral and anti-inflammatory effects of certain antidepressants in Covid-19” is added. (Page 10: lines 401-403)
- “the magnitude of the SSRIs in vitro inhibition of ASM” is added.(Page 10 and 11: lines 404-407)
- “Correlate of the magnitude of the in vitro antiviral effect in severity of disease” is added. (Page 11: lines 406-407)
- “A retrospective cohort study of an adult psychiatric facility operated in the New York State Office of Mental Health” is added. (Page 11: lines 437-439)
- The comment about “FIASMA antidepressant at baseline had a significantly reduced risk of intubation or death compared with those taking a non-FIASMA” is added. (Page 11: lines 439-442)
- “Four studies found that plasma ceramide levels strongly correlate with clinical and inflammation severity among these studies” are added (Page 10: lines 369-378)

13) Thanks for your fair and constructive comment. Discussion is corrected in regard of your suggestion. (Page 12: lines 468-476)

14) Thanks for pointing this out. Your suggested mechanism is added. (Page 12: lines 479-489)

15) Thanks for your recommendation. These two terms were replaced by your suggested terms.

16) Thanks for your suggestion. Conclusion is corrected. (Page 14: L545-548, page 15: lines 557-571)

**Reviewer 2 supplementary comments**

1) Thanks for your suggestion. Abbreviations are removed from abstract.

2) Thanks for pointing this out. Our included studies were in three study designs (clinical trials, case controls and cohorts) so we were inevitable to use three different tools for assessing their quality. But as we have mentioned in quality assessment section, among 12 included studies only three of them had high risk of bias so the total quality of included studies is appropriate.

3) Thanks for your fair and constructive comment. Your mentioned values are added into analysis results. (Page 7: lines 273-275)

4) Thanks for pointing this out. The I2 was lower than 50% so we used fixed model. The statistically analysis section of method part is corrected and this value is added. (Page 4: line 155)

5) Thanks for pointing this out. As its not correct to mix apples and oranges in meta-analysis we didn’t perform analysis for other drugs in different study types and only analyzed the effect of fluvoxamine in clinical trials.
